# Sex Differences in Mouse Popliteal Lymph Nodes

**DOI:** 10.1038/s41598-018-37175-5

**Published:** 2019-01-30

**Authors:** Riva Dill-Garlow, KuanHui Ethan Chen, Ameae M. Walker

**Affiliations:** Division of Biomedical Sciences, School of Medicine, University of California, Riverside, Riverside, CA 92521 USA

## Abstract

Females have more robust immune responses than males, well-illustrated by the degree of inflammation elicited during delayed-type hypersensitivity (DTH) responses. Here, we have investigated underlying sex differences that may contribute to differential footpad DTH responses using wildtype and four core genotypes (FCG) mice and popliteal lymphnode cellularity and gene expression. DTH responses in XX and XY FCG females showed no role for almost all genes expressed on sex chromosomes. After then filtering-out genes differentially expressed between XX and XY females, only one gene was sexually differentially expressed in wildtype mice, glycosylation-dependent cell adhesion molecule 1 (Glycam1), expressed 7-fold higher in females. Glycam1 facilitates leukocyte entry through high endothelial venules. Consistent with greater Glycam1 expression, female nodes contained twice as many cells. While females had more memory T cells in their nodes, males had a higher percentage of T regulatory cells. This sexual dimorphism in wildtype animals manifested pre-pubertally, was enhanced post-pubertally, and was eliminated by castration. The formation of male gonads is determined by the expression of Sry. Sry overexpression, which does not affect testosterone levels, produced an exaggerated male phenotype. We conclude that Sry expression through formation of the male gonad indirectly negatively impacts the potential for local inflammation.

## Introduction

Generally, females are considered to have stronger cell-mediated immune responses compared to males. For example, studies have reported the mortality rate from tuberculosis in women as half that in men^1,2^. When comparing the sexes for their ability to spontaneously clear Hepatitis C virus during acute infection, a process known to be reliant on cytotoxic CD8+ T-cells^3^, women were found to be more likely to clear the virus^4^. Experimentally, others have shown that female mice survive longer than males when infected with *Mycobacterium marinum*^5^. Although these and other studies provide documentation of sexual dimorphism in T cell-mediated immune responses, relatively little is known about causal factors for these acute responses or differences between the sexes that exist at steady state.

In a previous publication, we showed that female mice exhibit larger T cell-mediated delayed-type hypersensitivity (DTH) responses when compared to male mice^6^. However, contrary to expectations, ovariectomy increased, and estradiol replacement suppressed, the response. Also, others determined that administration of testosterone to castrated mice had only minimal suppressive effects on DTH responses^7^. Thus, reversible effects of the sex steroid hormones do not seem to be key to the greater DTH responses in females. Other effects of sex steroids can occur pre-pubertally and are permanent^8^ and these have not been examined in the context of DTH responses. It is also possible that additional factors, such as differential expression of genes on the X and Y chromosomes, could be important.

A model system that allows a determination of the separate roles of gonadal hormones and almost all X and Y genes is the Four Core Genotypes (FCG) mouse. In this model, the *Sry* gene, which is responsible for development as a male, has been deleted from the Y chromosome (Y-) and reintroduced as a transgene onto an autosome. This allows *Sry* to segregate independently from the Y- chromosome. By breeding these males (XY-*Sry*+) to wildtype XX females, four offspring genotypes are produced: XY-*Sry*+ (abbreviated XYM), XX*Sry*+ (abbreviated XXM), which are both gonadally male, and XY- (abbreviated XYF) and wildtype XX (XXF), which are both gonadally female^8^. By comparing XXM with XXF one can determine the role of gonadal hormones, and by comparing XYF with XXF one can determine the role of genes found on the sex chromosomes. This latter is true for all genes except *Sry* since maleness is absolutely dependent on the presence of *Sry*.

More recent analysis of the model has determined that there are 12–14 copies of the *Sry* gene inserted onto chromosome 3^9^. As a result, Sry is overexpressed in FCG male tissues (XYM and XXM) compared to wildtype males of the same strain. The increased copy number and overexpression of the gene provided a means to study the effects of *Sry*. Importantly for later discussion of results, although *Sry* is overexpressed in the male FCG mice, there is no difference in circulating testosterone levels between adult FCG and wildtype males^10^.

We report substantial differences in numbers of total cells, memory T cells, and T regulatory cells in the lymphnodes of wildtype males and females and that neither organizational effects of the sex steroids nor the expression of almost all genes on the sex chromosomes contribute to this sex difference. Rather, the sex difference is the indirect result of Sry expression in male gonads.

## Results

**Figure 1:**
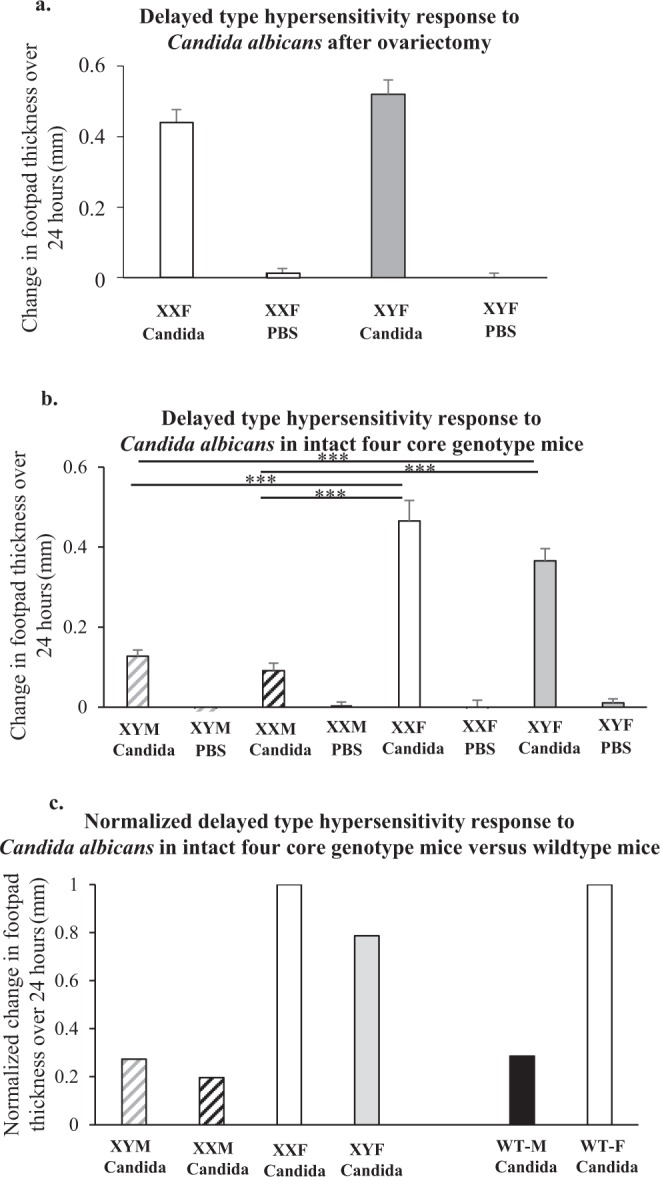
Delayed-type hypersensitivity response to *Candida albicans*. Data show the change in footpad swelling in mm over 24 hours for mice sensitized with *Candida albicans* (ovariectomized FCG-XXF n = 9, and ovariectomized FCG-XYF n = 10 (**a**)), (FCG-XYM n = 20, FCG-XXM n = 26, FCG-XXF n = 18, FCG-XYF n = 24 (**b**))or DPBS (ovariectomized FCG-XXF n = 11, and ovariectomized FCG-XYF n = 9 (**a**)), (FCG-XYM n = 10, FCG-XXM n = 22, FCG-XXF n = 6, FCG-XYF n = 16 (**b**)) and challenged with *Candida albicans* purified protein. Panel c is all FCG data normalized to FCG-XXF = 1, and wildtype B6 male normalized to wildtype B6 female = 1. Mann-Whitney statistical analysis was performed and significance was designated as follows: *P < 0.05, **P < 0.01, ***P < 0.001.

A footpad DTH response involves a major influx of cells, many of which come via lymphnodes in the region. Cells infiltrating the challenge site include NK cells, neutrophils and T cells^11^. Given the increased T cell mediated DTH response to *Candida albicans* in female compared to male mice, we asked whether female and male lymphnodes differed in their expression of genes relevant to cell recruitment. Because males and females possess different sex chromosomes and have many genes that differ in expression as a result, there was potential in this analysis for a significant amount of distracting information. To focus the analysis, we asked whether there was a difference in the DTH response between ovariectomized XX (wildtype) females and FCG XY- females. i.e. between phenotypically female animals with a different sex chromosome complement. As shown in Fig. 1a, there was no difference in the DTH response, allowing us to conclude that almost all genes coded for on the sex chromosomes were not relevant to the male/female difference in the DTH response. These genes could therefore be filtered out from among those that differed between wildtype male and female popliteal lymphnodes. For context, also shown in Fig. 1b,c are DTH responses in XY and XX FCG males and wildtype mice. This confirms previously published data illustrating the male-female difference and also that the FCG male phenotypes have a lower response than wildtype mice.

### RNA sequencing of lymph nodes

Sequencing of RNA from lymphnodes of intact wildtype males and females, as well as FCG XY and XX females, showed differential expression of only 1 gene after filtering out those differentially expressed between XXF and XYF. This was glycosylation dependent cell adhesion molecule 1 (Glycam1). Females expressed 7-fold higher levels of this gene (Table 1). Glycam1 expression is important to function of the high endothelial venules of lymph nodes^12^ Glycam1 supports T cell rolling in shear flow^13^ and binds to CD62L (L-Selectin), an adhesion molecule expressed on lymphocytes and important for their entry into secondary lymphoid tissues^14^.

**Table 1:** RNAseq analysis of GlyCAM-1 expression in lymphnodes of 5-month old wildtype males and females and males overexpressing Sry (SryHi).

GlyCAM-1	Fragments Per Kilobase of transcript per Million mapped reads
WT Female	1098
WT Male	129
SryHi Male	*90

*Not statistically different from wildtype males. N = 6 animals per category for WT Female and WT Male. N = 3 animals for SryHi Male.

### Sexual dimorphism in lymphnode cellular content in wildtype mice

**Figure 2:**
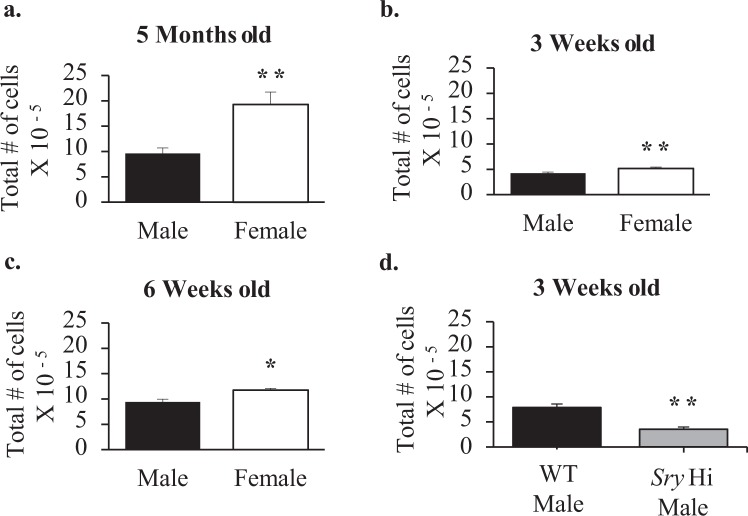
Total number of cells in the popliteal lymph nodes of wildtype and Sry Hi mice. Results shown are the average total number of cells counted per two popliteal lymph nodes of wildtype males and females at (**a**) 5 months of age (Male n = 4 Female n = 4), (**b**) 3 weeks of age (Male n = 4 Female n = 4), and (**c**) 6 weeks of age (Male n = 4 Female n = 4). Panel d compares wildtype (n = 5) and Sry Hi (n = 3) males at 3 weeks of age. Mann-Whitney statistical analysis was performed and significance was designated as follows: *P < 0.05, **P < 0.03, ***P < 0.001.

Given the sex-based difference in Glycam1 expression, we asked whether higher Glycam1 expression in females was associated with an increase in cellularity in the lymphnode in the absence of any local immune challenge. Popliteal lymphnodes from five-month old females were larger and had twice as many cells as in males (Fig. 2a). Male mice at 5 months of age weighed 28.5 ± 1.6 grams and females weighed 22.3 ± 1.6 grams. The data in Fig. 2a are not normalized, but normalization to body weight would amplify the sex difference. Analysis of glycam1 peptidoglycan released from popliteal nodes into medium showed the average amount released by female nodes (309.43 ± 200.2 pg/ml) to be 3.5 fold the average released by male nodes (89.3 ± 55.2 pg/ml). However, even though the trend was consistent with the mRNA data, this difference was not statistically significant.

### Lymphnode cell number sexual dimorphism as a function of maturity

**Figure 3:**
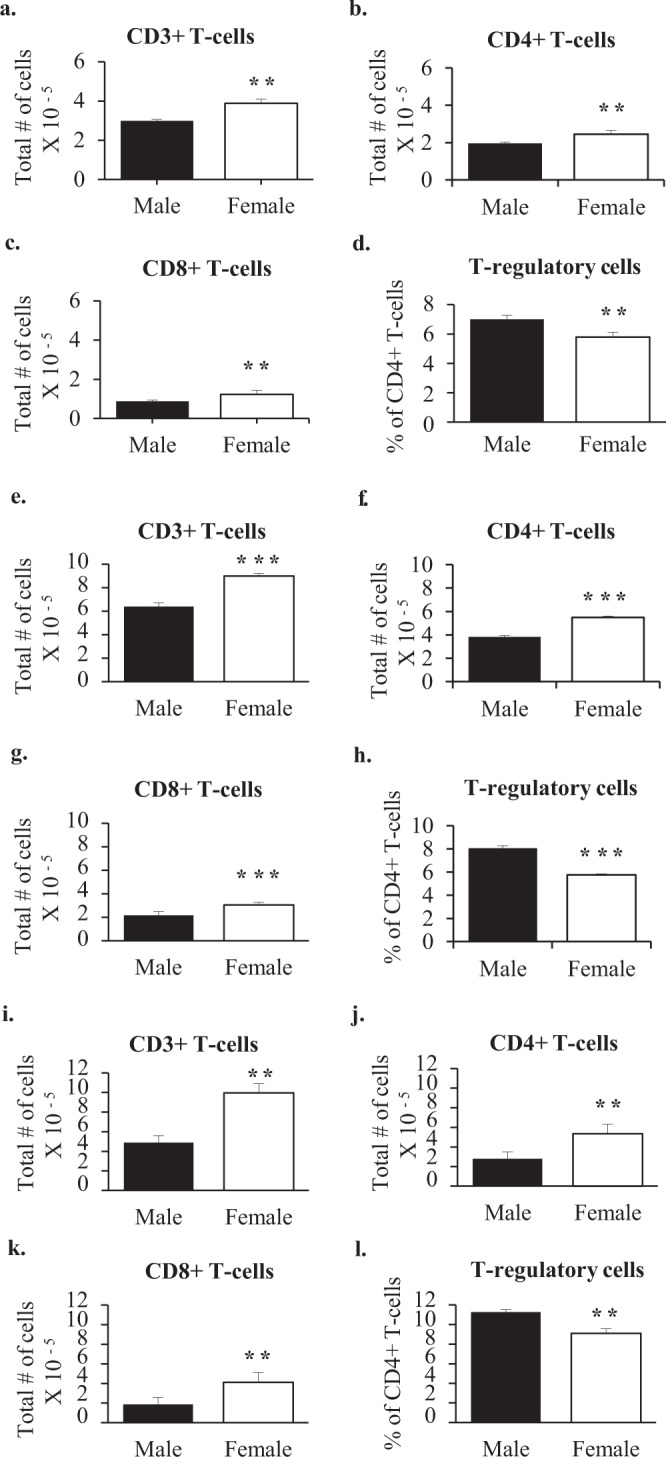
T cells in the popliteal lymph nodes of 3 week old, 6 week old, and 5 month old wildtype mice. Shown are the average total numbers of T cells, CD4+ T cells, CD8+ T cells, and % of CD4+ T cells that were T regulatory (CD25+/FOXP3+) per two popliteal lymph nodes of 3- week old (Male n = 4 Female n = 4) (**a**–**d**), 6-week old (Male n = 4 Female n = 4) (**e**–**h**), and 5-month old (Male n = 4 Female n = 4) (**i**–**l**) males and females. Mann-Whitney statistical analysis was performed and significance was designated as follows: *P < 0.05, **P < 0.03, ***P < 0.01.

Although in mice the first vaginal cornification, an indication of ovulation, does not occur until 34–36 days of age^15^, female mice are physiologically affected by circulating estrogens as early as 24 to 26 days of age, as evidenced by vaginal opening^16^. We therefore chose 21 days of age to determine whether there were any pre-pubertal sex differences in the popliteal lymphnodes. Even at this age, mice exhibited sex differences. Three-week old female mice had 25% more cells than males (Fig. 2b). Among these, there were 20% more T cells (CD3+), 15% more CD4+ T cells and 30% more CD8+ T cells than males (Fig. 3a–c). Three-week old males also had a 20% higher percentage of CD4+ cells that were T regulatory (CD25+/FOXP3+) (Fig. 3d).

At 6 weeks of age, the sex difference in the popliteal lymph nodes was increased. Although female mice still had 25% more cells (Fig. 2c), among these there were now 40% more T cells, 45% more CD4+ T cells, and 40% more CD8+ T cells (Fig. 3e–g). Males had an even higher percentage of CD4+ cells that were T regulatory, i.e. a 40% higher proportion than that observed in females (Fig. 3h).

At 5 months of age, the sexual dimorphism in the popliteal nodes increased still further. Females had 105% more T cells (CD3+), 90% more CD4+ T cells, and 120% more CD8+ T cells. Males maintained a higher percentage of CD4+ cells that were T regulatory, although the relative difference compared to the proportion of T regulatory cells in females decreased from 40% to 20% (Fig. 3i–l).

### Pre-pubertal sexual dimorphism is regulated by *Sry*

**Figure 4:**
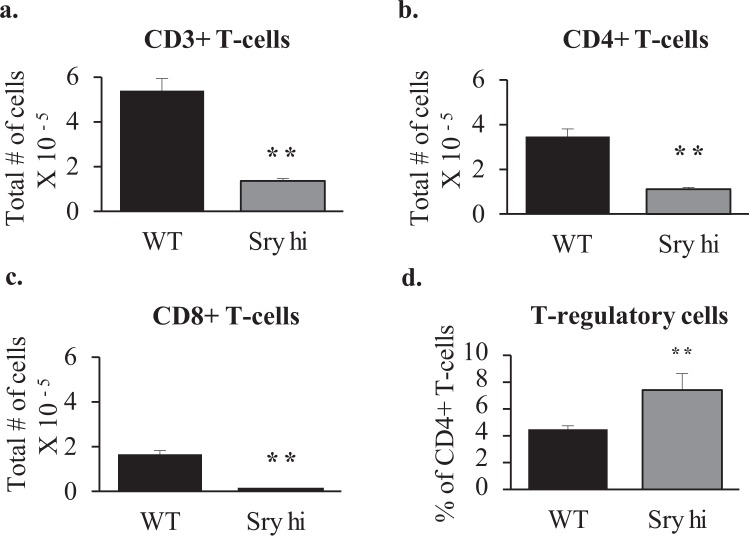
T cells in the popliteal lymph nodes of wildtype males and *Sry* overexpressing males at 3 weeks of age. Shown are the average total numbers of (**a**) T cells, (**c**) CD4+ T cells (**c**) CD8+ T cells, and (**d**) % of CD4+ T cells that were T regulatory (CD25+/FOXP3+) per two popliteal lymph nodes of wild type males (WT n = 5) and *Sry* overexpressing males (*Sry* Hi n = 3). Mann-Whitney statistical analysis was performed and significance was designated as follows: *P < 0.05, **P < 0.03, ***P < 0.01.

Although use of the two female genotypes in the FCG model allowed us to eliminate most genes on the X and Y chromosomes as being important to sexual dimorphism in the DTH response, there is one gene on the Y chromosome, *Sry*, that is responsible for producing a male phenotype and hence cannot be isolated from other aspects of maleness. To determine whether Sry expression contributed to the pre-pubertal establishment of sexual dimorphism in the popliteal lymph node, we compared wildtype males with males overexpressing Sry (FCG XY-*Sry*+) at 3 weeks of age. At this age, wildtype males had twice as many cells in their popliteal lymph node compared to Sry overexpressing males (Fig. 2d). There were 4 times as many T cells, 3 times as many CD4+ T cells, and 15 times as many CD8+ T cells in wildtype males (Fig. 4a–c). Sry over-expressing males also had 65% higher percentages of CD4+ cells that were T regulatory cells (Fig. 4d). However, overexpression of Sry did not produce a significant change in the level of expression of Glycam1, although the trend was towards reduced expression (Table 1).

### Sexual dimorphism in wildtype young adults is regulated by male gonadal secretions

**Figure 5:**
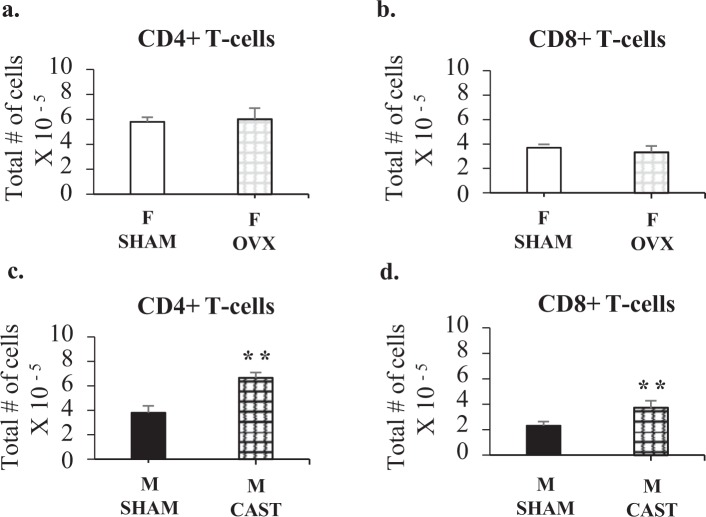
T cell numbers in the popliteal lymph node of 6-week old wildtype mice with either gonadectomy or sham surgery. Shown are the average total numbers of (**a**,**c**) CD4+ T-cells, (**b,d**) CD8+ T-cells per two popliteal lymph nodes of 6-week old females with sham surgery (F SHAM n = 5), females with ovariectomy (F OVX n = 5), males with sham surgery (M SHAM n = 5), or males with castration (M CAST n = 7) at 3 weeks of age. Mann-Whitney statistical analysis was performed and significance was designated as follows: *P < 0.05, **P < 0.03, ***P < 0.01.

The results in Figs 2 and 3 show that the degree of sexual dimorphism increased from 3 to 6 weeks of age. During this period, mice enter puberty and thus are exposed to adult levels of gonadal hormones^17^. To determine whether male or female gonadal hormones were responsible for the increasing dimorphism, gonadectomies or sham surgeries were performed on mice at 3 weeks of age and the cellular makeup of the popliteal lymph nodes determined at 6 weeks of age. CD4+ and CD8+ T cell numbers in the popliteal lymph nodes of ovariectomized mice were not different from sham operated female mice (Fig. 5a,b). By contrast, cell numbers increased in castrated mice to attain female numbers (Fig. 5c,d). This result tells us that the effect of Sry expression is mediated through effects on the gonads and not through direct effects on the lymphnode. This is particularly important for interpretation of the Sry overexpression result since overexpression can produce extraneous expression.

### CD62L Expression on CD4+ T cells and CD8+ T cells in wildtype mice

**Figure 6:**
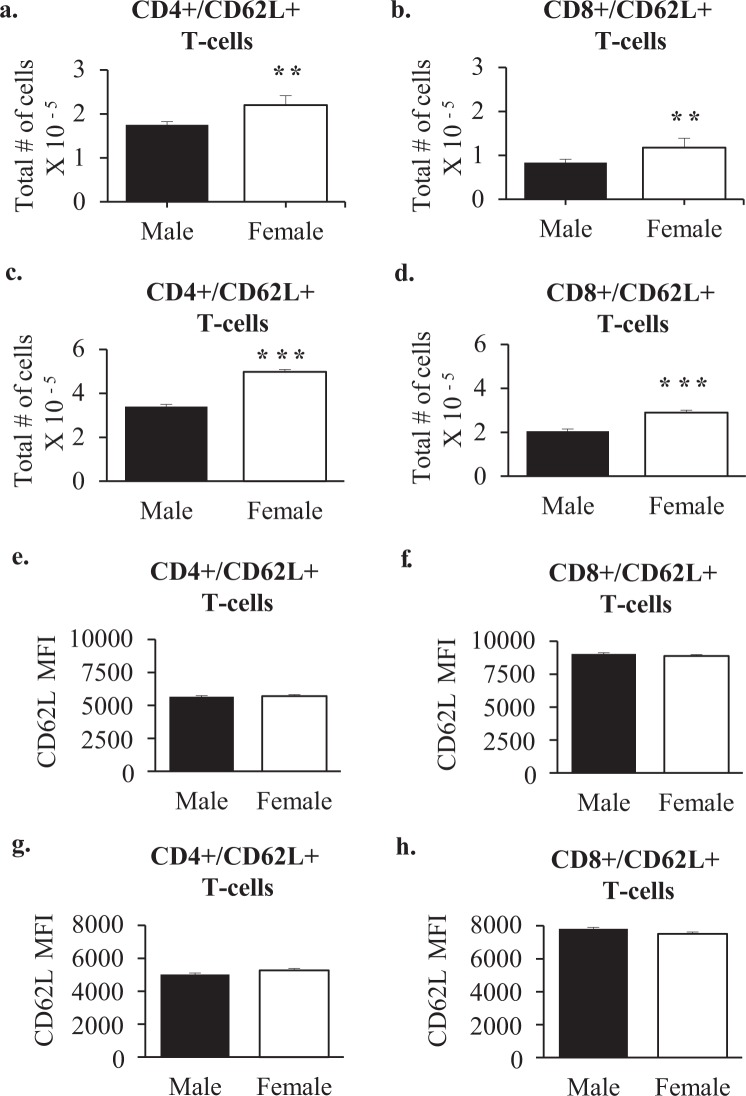
CD62L+ T cells in the popliteal lymph nodes of 3- and 6-week old wildtype mice. Shown are the average total numbers of CD4+/CD62L+ T cells and CD8+/CD62L+ T-cells, per two popliteal lymph nodes of 3-week old (Male n = 4 Female n = 4) (**a**,**b**) and 6-week old (Male n = 4 Female n = 4) (**c**,**d**) males and females, and CD62L Mean Fluorescence Intensity (MFI) on the same cell types in the popliteal lymph nodes of 3-week old (**e**,**f**) and 6 week old (**g**,**h**) males and females. Mann-Whitney statistical analysis was performed and significance was designated as follows: *P < 0.05, **P < 0.03, ***P < 0.01.

CD62L is a cell adhesion molecule that binds to Glycam1. Females had more CD4+ and CD8+ T cells that were also CD62L+ at both 3 (Fig. 6a,b) and 6 weeks of age (Fig. 6c,d). The degree of difference between males and females was very similar to the degree of difference in total CD4+ and CD8+ cells i.e. most CD4+ and CD8+ cells were also CD62L positive. At neither 3 nor 6 weeks of age did the level of CD62L expressed per CD4+ (Fig. 6e,g), or CD8+ T cell (Fig. 6f,h) differ on the basis of sex, as assessed by mean fluorescence intensity.

### Memory T cells in the popliteal lymphnode of wildtype mice

**Figure 7:**
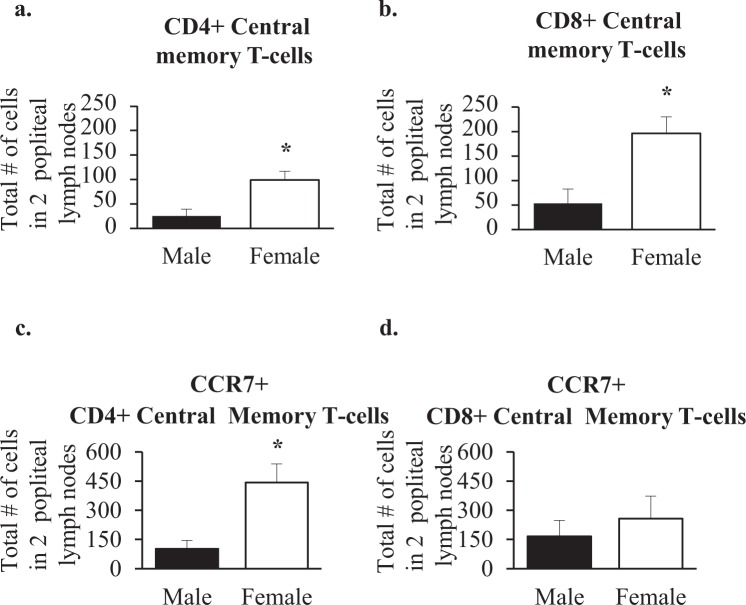
Central memory T cells in the popliteal lymphnode of wildtype mice. Shown are average numbers of CD4+ (**a**,**c**) and CD8+ (**b**,**d**) memory T cells per two lymph nodes of 5-month old male and female animals (Male n = 3 in A and B, and n = 5 in c and d, Female n = 3 in a and b, and n = 5 in c and d). Mann-Whitney statistical analysis was performed and significance was designated as follows: *P < 0.05.

Of relevance to an effective local response to any antigen challenge is the relative number of memory T cells. Females had about 5 times as many CD4+ central memory cells defined as (CD4+/CD44+/CD62L+)^18^ (Fig. 7a) and 4 times as many CD8+ central memory cells defined as (CD8+/CD44+/CD62L+)^18^ (Fig. 7b) than males. In a second set of experiments using CCR7 as an additional marker, there were about 4 times as many CD4+ central memory cells (Fig. 7c) and about 50% more CD8+ central memory cells (Fig. 7d), although the latter difference was not statistically significant. Thus, accumulation of central memory T cells in females was proportionally greater than for total T cells (compare with Fig. 3j,k) for both CD4+ and CD8+ using CD44 and CD62L as markers of central memory and for only CD4+ using the additional CCR7 surface marker.

### Sexual dimorphism in the inguinal lymphnode and the spleen of wildtype mice

**Figure 8:**
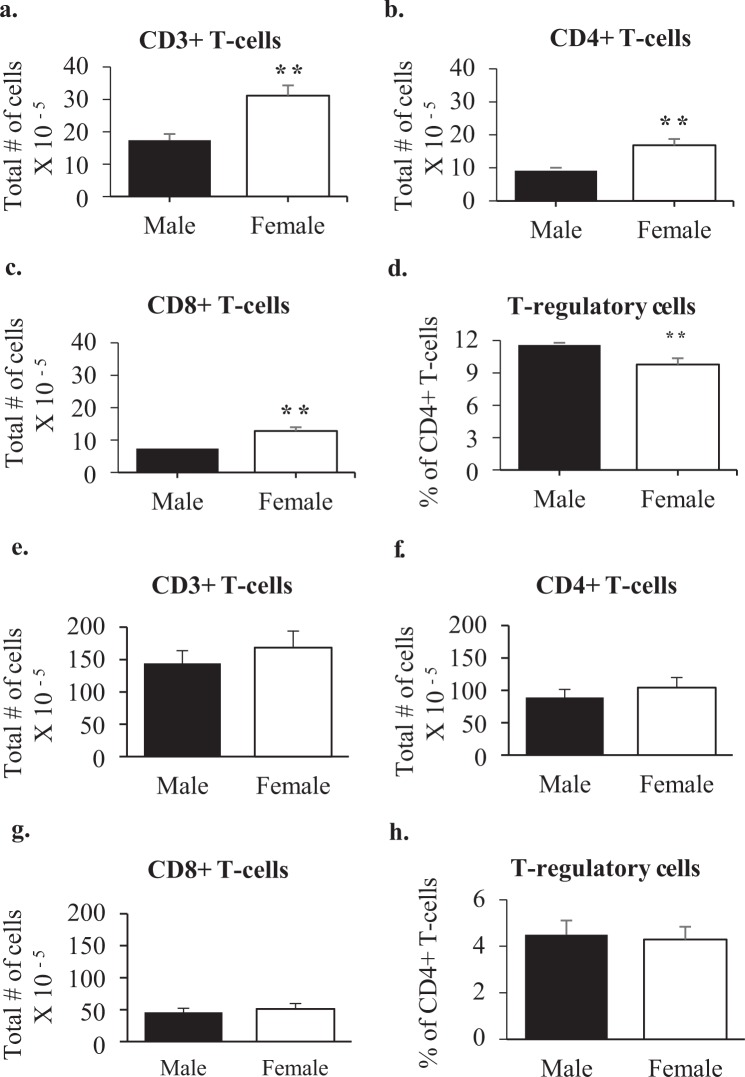
T cell numbers in the inguinal lymphnodes and spleen of 5-month old wildtype males and females. Shown are the average total numbers of T cells (**a**,**e**), CD4+ T cells (**b**,**f**), CD8+ T cells (**c,g**), and % of CD4+ T cells that were T regulatory (CD25+/FOXP3+) (**d**,**h**) per two inguinal lymph nodes (Male n = 4 Female n = 4) (**a**–**d**) and spleen (Male n = 4 Female n = 4) (**e**–**h**) of 5-month old males and females. Mann-Whitney statistical analysis was performed and significance was designated as follows: *P < 0.05, **P < 0.03, ***P < 0.01.

To determine whether the sexual dimorphism was specific to the popliteal lymphnode, similar analyses of the inguinal node were performed at 5 months of age. Figure 8 shows the results for CD3+ (A), CD4+ (B), and CD8+ (C) cells, which were all higher in females, and T regulatory cells (D), which were higher in males. i.e. a very similar pattern to the popliteal was observed for the inguinal lymphnode. By contrast, there was no sex difference in any of these measures in the spleen (Fig. 8e–h).

## Discussion

Delayed-type hypersensitivity responses to *Candida albicans* are T cell-mediated immune responses. Mice are sensitized by intradermal injection of *Candida albicans*. Antigen-presenting cells digest and display *Candida* peptides on MHC molecules to T cells. Presentation, in combination with induction of co-stimulatory molecules, activates T cells and leads to the development of specific memory T cells that then circulate and become distributed throughout tissues of the body. Upon challenge 7 days later, in this case through footpad injection of *Candida albicans* purified protein, antigen presenting cells carry processed and displayed *Candida* derivative peptides to the tissue-draining popliteal lymph nodes where they encounter the *Candida*-specific memory T cells, thereby precipitating an immune response. Activated T cells subsequently proliferate and travel to the site of challenge where they produce inflammatory cytokines and signal local endothelial cells to increase adhesion molecule expression and blood vessel permeability. Increased permeability allows plasma and circulating cells to exit the circulation, resulting in swelling of the tissue. Measurement of the inflammatory swelling indicates the robustness of the response^19^.

Previous work in the laboratory had shown that adult females have a larger response than males in both this Candida footpad and a skin contact hypersensitivity DTH assay^6^. Contrary to expectations at the time, ovariectomy increased, and administration of estradiol decreased, the responses, thereby illustrating that the increased female response was certainly not due to stimulatory effects of female gonadal hormones. Also, others had determined that administration of testosterone to castrated mice had only a minimal suppressive effect on DTH responses^7^. Thus, reversible effects of the sex steroid hormones did not seem to be relevant to the greater DTH responses in females. However, as mentioned in the introduction, some effects of sex steroids can occur pre-pubertally and are permanent^8^, and these had yet to be examined in the context of DTH responses. It was also possible that other factors, such as differential expression of genes on the X and Y chromosomes, could be important. To investigate these possibilities, we used the footpad DTH response and focused on the most important lymphnode involved, the popliteal^20^.

Differences in gene expression between males and females can arise from the expression of genes on the Y chromosome, from increased expression of genes on the X chromosome due to incomplete X inactivation, and from differences in the hormonal milieu^21^. To determine whether expression of genes on the X or Y chromosome played gonadal hormone-independent roles, we tested the DTH response in ovariectomized XXF and XYF animals. By determining that ovariectomized XXF and XYF had equivalent DTH responses, we were able to eliminate all but one of the sex chromosome genes (*Sry)* as playing a role in the differential DTH responses between males and females. This filtering process was extremely successful since only one gene remained, Glycam1.

Glycam1 has a number of functions, but in the lymphnode, where it is highly expressed, its major function is to bind CD62L on the surface of T cells^22–24^. Since female lymphnodes express around 7-fold higher levels of Glycam1 mRNA than male lymphnodes, they are equipped to encourage greater transmigration of T cells from the blood into the lymphnode. Measurement of Glycam1 peptidoglycan showed a similar trend, with the average of females being 3.5 fold the average in males. However, this difference was not statistically significant because of inter-animal variability or variability of elution into conditioned medium. Consistent with expectations based on Glycam1 expression, females maintained larger numbers of CD4+ and CD8+ T cells in their lymph nodes than males. There were no differences in CD62L expression per cell, and almost all T cells in the node expressed CD62L and so, at least for the CD62L-Glycam1 interactions, increased migration into the lymphnode in females correlates well with Glycam1 expression. Central memory T cells express CD62L and therefore home to lymphnodes^18^. There were more central memory T cells in female popliteal lymphnodes, again correlating with greater expression of Glycam1. There is no Glycam1 expression in the spleen^24^ where there were also no significant sex differences in T cell populations.

Having higher numbers of memory T cells in the female lymphnode increases the chance of there being an appropriate memory cell awaiting antigen presentation after challenge, thereby resulting in the opportunity for more robust responses in females.

T regulatory cells suppress immune responses, making their proportion in relation to other T-cells more demonstrative of their physiologic role rather than their total numbers^25,26^. By determining the percentage of CD4+ cells that were T regulatory, we found that males had a higher percentage in the node. Therefore, not only do males have fewer T cells in the node, but a larger proportion of them are capable of dampening a response. Both of these findings likely contribute to the less robust response in males.

At 3 weeks of age and therefore prior to the onset of puberty, sexual dimorphism of the popliteal lymph node was already in place, albeit to a smaller degree for CD4+ and CD8+ T cell numbers than was found in adults. This pre-pubertal sexual dimorphism is to some extent dependent on *Sry* expression since overexpression of Sry produced an exaggeration of the normal dimorphism, reducing T cell numbers over wildtype males and increasing the percentage of CD4+ cells that were T regulatory. However, exaggeration of the male cellular phenotype was not accompanied by a significantly lower level of expression of Glycam1, although the trend was towards lower expression in the Sry overexpressing animals. It therefore appears that Glycam1 is important, but may not be the only factor involved even though it was the only gene differentially expressed in lymphnodes between males and females. Since all gene expression is normalized, a change in lymphnode structural cell number, for example, would not be detected by the analyses performed.

In the mice overexpressing Sry, it could have been that extra-gonadal expression of Sry had a direct effect on expression of Glycam1 in the lymphnode. However, castration at 3 weeks completely feminized the number of cells in the popliteal lymphnode at 6 weeks, thereby demonstrating the effect of Sry expression on the lymphnode in both the wildtype and Sry overexpressing situation is indirect and a consequence of the presence of testes.

Sry expression is crucial to the development of the testes and the production of all testicular hormones^27^, including testosterone, activin and inhibin. In male, but not female, mice there is a perinatal surge of testosterone^28^, which has important permanent (organizational) masculinizing effects in a number of tissues^29,30^. However, the effect on the cellular content of the lymphnodes was reversible by castration at 3 weeks, demonstrating the sex difference in the lymphnode is not among the organizational effects of perinatal testosterone. This result does not preclude the existence of reversible, activational effects of testosterone, but given the exaggerated male phenotype in the Sry overexpressing FCG males, one would expect elevated testosterone levels in those mice if this were the case. However, measurement of testosterone levels in adult wildtype and FCG males showed no differences^10^, a finding we have confirmed in limited sampling of neonatal and adult mice in our own laboratory (unpublished data). Therefore, it seems likely that some other secretory product of the testes, such as activin or inhibin^31^, directly or indirectly affects the lymphnode.

Inhibin, which is produced by Sertoli cells in the testes, is required for normal maturation of dendritic cells^32^, which in turn positively regulate the function of high endothelial venules, including the expression of Glycam1^33^. Since inhibin production rises at puberty in both sexes^34^ and the effect of inhibin is to increase Glycam1, inhibin does not appear to be a good candidate for the crucial male factor. Rather, the data suggest that the male profile in our results appears equivalent to partial inhibin knockdowns^32^. Since activins and inhibins have generally counter-regulatory activities^35^, this leads us to our current working hypothesis that the male phenotype is caused by increased production of activin or some other effect that mimics inhibin knockdown. While an attractive hypothesis, the complexity of the activin-inhibin family, activin-inhibin interactions, binding proteins and receptors and effects on the pituitary, means it will take some time to prove or disprove this working hypothesis.

Because our original observations showed sex differences in the footpad DTH response, we focused analyses on the popliteal lymphnode. However, similar sex differences were seen in the adult inguinal lymphnode in regard to T cell numbers and previous work has shown DTH responses in the skin of the ear were also greater in females^6^. Therefore, it seems likely that lymphnodes in general contain more effector and/or fewer T regulatory cells in females compared to those in males. Only one other study has found any sexual dimorphism by measuring T cell numbers, in this case determining adult females had more T cells in the peritoneal and pleural cavities than adult males^36^. However, this study did not assess pre-pubertal sex differences or the relative role of male gonadal secretions. Instead, they focused on the function of female gonadal hormones and were unable to explain the sex differences in T cell numbers by the presence or absence of female gonadal hormones. This agrees quite well with the general finding in the current study that it is the presence of male gonads that creates the sex difference.

While the current study has focused on one aspect of sex differences of relevance to cell-mediated immunity, there are also sex differences in antibody-mediated immune responses and susceptibility to antibody-mediated autoimmune diseases^37^. It is also known that at least some strains of mice exhibit sexual dimorphism of their gut microbiota^38,39^, and the sex-dictated microbiota is capable of influencing not only hormone levels, but also susceptibility to autoimmune disease and general immune function^39–41^. What we have described in the current manuscript is therefore only one part of the sex differences found in immune responses and may even be only one aspect of sex differences important to the very complex DTH response. Nevertheless, it is an intriguing and substantial sex difference.

In summary, the presence of more effector and memory cells and a smaller proportion of T regulatory cells in the female lymphnode creates the potential for larger DTH responses. The mechanism through which female lymphnodes accumulate more effector cells is most likely via greater expression of Glycam1 on high endothelial venules of the lymphnodes. The male phenotype is fully reversible by gonadectomy, and although testosterone may contribute, there is evidence for an important role for other male gonadal secretions. The very different effector and regulatory cell contents of the lymphnodes between the sexes further highlights the importance of examining sex as a variable in analysis of disease progression or treatment responsiveness.

## Methods

### Animals

Animals were housed in a specific pathogen free facility on a 12-hour light/dark cycle. All animal procedures were approved by the University of California, Riverside, Institutional Animal Care and Use Committee and were in accordance with guidelines from the American Association for Laboratory Animal Care. Adult male and female C57Bl/6J mice and Four Core Genotypes (FCG) XY^− +*Sry*^ (C57Bl6/J background) male mice were obtained from Jackson Laboratories and bred to produce the different ages and genotypes investigated. FCG offspring were genotyped using the protocol recommended by Jackson Laboratories.

### Animal Procedures

Delayed-Type Hypersensitivity Responses to *Candida albicans* were initiated by intradermal injection in both flanks of 10 × 10^6^ formalin-fixed cells in a volume of 100 µL Dulbecco’s phosphate buffered saline (DPBS) using the same volume of DPBS as control. Seven days later, footpads were measured by caliper for baseline values, and 50 µL of *Candida albicans* purified antigen (1.4 mg/mL) (Alerchek, Portland ME) were injected into each footpad (in both experimental and control animals). Twenty four hours later, footpads were measured and the average footpad swelling in each animal recorded.

The spleen, both popliteal and both inguinal lymph nodes were collected, the two popliteal or inguinal lymphnodes from each mouse were combined and all tissues gently pressed through sterile 70 µm cell strainers (Corning 352350 Manassas, VA) while suspended in RPMI1640. Cell number was measured using a Cell Scepter (EMD Millipore, Billerica, MA) with a 40 µm sensor.

Gonadectomies or sham surgeries in wildtype mice occurred at 21 days of age, with subsequent experimentation at 6 weeks of age. Ovariectomies of XXF and XYF mice occurred at 10 weeks of age with testing of DTH responses 4 weeks later.

### Flow cytometric analysis

**Figure 9:**
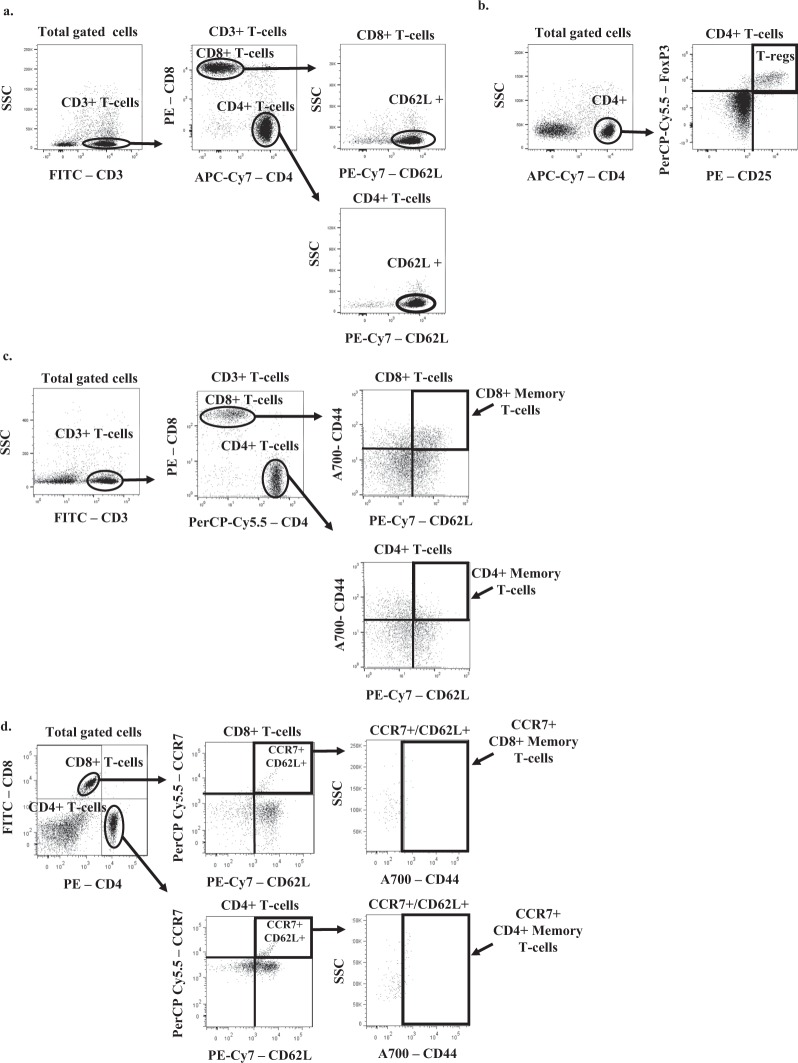
Representative gating strategies used to analyze cell types by flow cytometry. Shown are representative dot plots with gating for CD4+/CD62L+ and CD8+/CD62L+ T-cells (**a**), T-regulatory Cells (CD4+/CD25+/FOXP3+) (**b**), CD4+ Memory T-cells (CD4+/CD44+/CD62L+) and CD8+ Memory T-cells (CD8+/CD44+/CD62L+) (**c**), and CCR7+/CD4+ Memory T-cells and CCR7+/CD8+ Memory T-cells (**d**).

All antibodies were from eBioscience, San Diego, CA, diluted in 0.2% BSA in DPBS (FACS buffer). Fc receptors were blocked by incubation in purified anti-CD16/32 (14-0161) diluted 1:50 for 15 minutes at 4 °C. Cells were then washed with FACS buffer, and resuspended in a mixture of fluorescently-labeled antibodies: 0.5:100 rat anti-mouse CD3 (11-0031), rat anti-mouse CD4 (47-0041), rat anti-mouse CD8 (17-0081), rat anti-mouse CD25 (12-0251), rat anti-mouse CD44 (56-0441), rat anti-mouse CCR7 (45-1971), and rat anti-mouse CD62L (25-0621), or appropriate isotype controls for 1 hour at 4 °C in the dark. For T regulatory cell staining, after Fc block and extracellular staining with anti-CD4 and -CD25, intracellular staining with 0.6:100 rat anti-mouse FoxP3 (45-5773) was performed using an intracellular staining set (00-5523) per manufacturer’s instructions. Most cells were washed, resuspended in FACS buffer and run live on the BD Facs Canto II. T regulatory cell staining, was of fixed and permeabilized cells. FlowJo software was used for analysis, and gates were determined based on either isotype control staining or fluorescence minus one controls. Dot plots of representative gating strategies used in this manuscript are shown in Fig. 9.

### RNA sequencing, library preparation and analysis

Pooled RNA (*n* = 3–6 or 6 animals per group depending on whether an FCG genotype or wildtype, respectively) was used to make libraries. Whole tissues (parenchyma and stroma) were homogenized and RNA was extracted by Trizol-Chloroform phase separation. RNA was then purified and concentrated using Qiagen Rneasy Mini columns (Valencia, CA). The same amount of total RNA for each sample was used to prepare libraries using the Kapa Biosystems Stranded library preparation kit with Riboerase (Wilmington MA). Libraries were labeled with barcodes from a New England Biosciences kit (Ipswich MA). After library preparation, samples were assessed for quantity and quality by bioanalyzer. Equal amounts of each library were multiplexed and submitted to the facility at University of California, San Francisco, Parnassas, CA for sequencing by Rapid paired end 2 × 50 on the Hiseq2500. Sequencing was per Illumina (San Diego, CA) protocol in rapid run mode. Samtools were used to create bam files^42,43^ to be mapped to the mouse genome using HiSat2^44,45^. Cufflinks was used to assemble transcriptomes from RNA-Seq data and quantify expression. Differentially expressed genes were determined by cuffdiff^46,47^. Only genes with differential expression greater than 2-fold were considered meaningfully different.

### Glycam1 peptidoglycan measurement

Glycam1 is only relatively loosely associated with membranes of the high endothelial venules and incubation of disrupted lymphnodes has been shown to result in movement of Glycam1 into medium^48^. Both popliteal nodes from each animal were used for collection of lymphocytes, as above, and further incubated in 1 ml RPMI 1640 for 2 hours at 37 °C. Cells were washed through the filter with a further 0.5 ml RPMI, pelleted and used for flow cytometry while the supernatant was used to determine Glycam1. Mouse Glycam1 was assayed using a kit obtained from Abbexa (Cambridge, UK). The results are expressed as per ml of conditioned medium.

### Other Statistical Analyses

Statistical significance for non-RNAseq data was determined using the Mann-Whitney test. Sample sizes are indicated in figure legends and data are presented as mean ± Standard Error. *P*-values were designated as follows throughout the manuscript; *P < 0.05, **P < 0.03, ***P < 0.01.

## Data Availability

RNAseq data files used to generate Table 1 have been deposited to NCBI’s Gene Expression Omnibus and are accessible through GEO Series accession number (GSE98073). Private access link: https://www.ncbi.nlm.nih.gov/geo/query/acc.cgi?token=ityjeausjtcdzmh&acc=GSE98073.
